# Habitual exercise decreases plasma xanthine oxidoreductase activity in middle-aged and older women

**DOI:** 10.3164/jcbn.17-101

**Published:** 2018-03-30

**Authors:** Keisei Kosaki, Atsuko Kamijo-Ikemori, Takeshi Sugaya, Koichiro Tanahashi, Nobuhiko Akazawa, Chihiro Hibi, Takashi Nakamura, Takayo Murase, Kenjiro Kimura, Yugo Shibagaki, Seiji Maeda

**Affiliations:** 1 Graduate School of Comprehensive Human Sciences, University of Tsukuba, Ibaraki 305-8577, Japan; 2 Division of Nephrology and Hypertension, Department of Internal Medicine, St. Marianna University School of Medicine, Kanagawa 216-8511, Japan; 3 Department of Anatomy, St. Marianna University School of Medicine, Kanagawa 216-8511, Japan; 4 CMIC Holdings Company Ltd., Tokyo 105-0023, Japan; 5 Faculty of Health and Sport Sciences, University of Tsukuba, Ibaraki 305-8574, Japan; 6 Japan Institute of Sports Sciences, Tokyo 115-0056, Japan; 7 Biopharmaceutial Study Group, Pharmaceutical Research Laboratories, Sanwa Kagaku Kenkyusho Company Limited, Mie 511-0406, Japan; 8 Radioisotope and Chemical Analysis Center, Sanwa Kagaku Kenkyusho Company Limited, Mie 511-0406, Japan; 9 JCHO Tokyo Takanawa Hospital, Tokyo 108-8606, Japan

**Keywords:** cardiovascular disease risks, visceral fat, daily step counts, aerobic exercise training

## Abstract

The aim of present study was to investigate the association between plasma xanthine oxidoreductase activity, which has gained attention as a novel preventive target of cardiovascular disease, and various physiological parameters and was to determine the effects of habitual exercise on plasma xanthine oxidoreductase activity in middle-aged and older women. In the cross-sectional study, we investigated the association between plasma xanthine oxidoreductase activity and various physiological parameters in 94 middle-aged and older women. In the interventional study, subjects (*n* = 22) were divided into two groups: exercise (*n* = 12) or the control group (*n* = 10), whereby we examined the effect of 12-week aerobic exercise training on plasma xanthine oxidoreductase activity in middle-aged and older women. The cross-sectional study demonstrated that plasma xanthine oxidoreductase activity was significantly associated with various physiological parameters, including visceral fat and daily step counts. In the interventional study, the plasma xanthine oxidoreductase activity significantly decreased after the 12-week aerobic exercise training, its changes were inversely associated with the changes in daily step counts. Our results revealed that the plasma xanthine oxidoreductase activity was associated with visceral fat accumulation and lack of exercise, and it was decreased by the aerobic exercise training.

## Introduction

Cardiovascular disease (CVD) risks, including arteriosclerosis and endothelial dysfunction, have been demonstrated to increase with aging.^(1,2)^ In addition to the age-associated risks, the middle-aged and older women carry further CVD risks by the influence of various physiological changes with menopause.^(3,4)^ Therefore, it is clinically important to reduce the CVD risks and to suppress the onset of CVD in middle-aged and older women.

Xanthine oxidoreductase (XOR) is expressed in various organs including the liver, lung, heart and kidney, vascular (i.e., endothelial cell) and adipose tissues,^(5)^ and the activity of XOR is particularly higher in visceral adipose tissues.^(6)^ XOR is known as a representative enzyme for uric acid production (i.e., the purine metabolism) and generally functions as xanthine dehydrogenase (XDH);^(7)^ the protein structure of XOR is converted to xanthine oxidase (XO) under the tissue hypoxic condition, which induces the production of reactive oxygen species.^(8)^ Therefore, the increase in XOR activity during such pathological conditions is likely to be associated with the onset and aggravation of various chronic diseases. It has previously been reported that higher levels of plasma XOR activity are closely associated with clinical outcomes, including cardiac events and mortality, in patients with chronic heart failure.^(9)^ Similarly, previous studies have demonstrated that the plasma/serum activity of XOR was higher in various disease states, including coronary artery disease and non-alcoholic fatty liver disease, compared with that of healthy control subjects.^(10,11)^ Therefore, the increase in XOR activity is regarded as one of the pathophysiological factors leading to the onset of CVD and is regarded as a potential novel preventive target.^(12,13)^ However, to the best of our knowledge, there are no previous studies investigating XOR activity in middle-aged and older women.

Lifestyle modification, particularly habitual exercise, has been endorsed as an effective strategy for reducing the CVD risks in various disease populations, as well as in the general population.^(14,15)^ Several previous studies have reported that habitual exercise decreased blood pressure and increased arterial compliance, endothelial function and nitric oxide production (i.e., decreased CVD risks).^(16–21)^ Additionally, habitual exercise has been reported as an effective method to improve obesity-associated variables, including visceral fat in obese individuals.^(22)^ Based on these findings, it has been suggested that habitual exercise may decrease visceral fat and subsequently reduce XOR activity and the risks of CVD in middle-aged and older women; however, the effects of habitual exercise on XOR activity remain to be elucidated.

The aim of this study was firstly to investigate the association between plasma XOR activity and various physiological parameters, and secondly to determine the effects of habitual exercise on plasma XOR activity in middle-aged and older women. To comprehensively achieve these aims, we used two different protocols. Protocol 1 (cross-sectional approach) was designed to determine the association between plasma XOR activity and various physiological parameters. Protocol 2 (interventional approach) was designed to evaluate the effect of aerobic exercise training on plasma XOR activity.

## Materials and Methods

### Subjects

For protocol 1, 94 middle-aged and older women (aged 50–74 years) were included in the cross-sectional study. For protocol 2, 22 middle-aged and older women (aged 51–72 years) participated in the interventional study. The subjects were divided into two groups: a control group (*n* = 10) and an exercise group (*n* = 12). The present study only allocated participating individuals who wished to participate in aerobic exercise training to the exercise group in order to keep the continuation rate as high as possible. Throughout the protocol, all subjects were free from cardiovascular and renal diseases, as assessed by their medical records. The present study was approved by the Ethical Committees of the Institute of Health and Sport Sciences of the University of Tsukuba (IRB approval number; Tai 25-127 and Tai 26-122). The present study was performed in accordance with the principles outlined in the Declaration of Helsinki, and all subjects provided written informed consent prior to enrolment in the present study.

### Procedures

All evaluations were performed in the morning after a 12-h overnight fast. Evaluations were obtained in a quiet, temperature-controlled room (24–26°C). Following a resting period of ≥20 min, the hemodynamic parameters were determined and blood samples were obtained to determine the blood biochemistry. For protocol 2, these evaluations were performed before and after the 12-week intervention.

### Aerobic exercise training

The subjects in the exercise group underwent aerobic exercise training for ≥3 days/week for 12 weeks. The training program consisted of 2–3 days/week of supervised bicycling and walking exercises at the University of Tsukuba and additional home-based aerobic exercise training, as previously described.^(23)^ The subjects initially performed the supervised aerobic exercise training of bicycling or walking 30 min/day at a relatively low intensity of exercise (60% of their individually determined maximal heart rate). As the subjects’ exercise tolerance improved, the aerobic exercise training was increased to 40–50 min/day at an intensity of 65–80% of their maximal heart rate. Subjects in the control group were instructed not to alter their baseline physical activity levels.

### Clinical measurements

Body weight was determined to the nearest 0.1 kg using a digital scale. Height was measured to the nearest 0.1 cm using a wall-mounted stadiometer. Body mass index was calculated as the subjects’ weight (kg) divided by their height (m^2^). Visceral fat was evaluated using the dual-impedance analysis method (HSD-2000; Omron Healthcare, Kyoto). Waist circumference was measured in duplicate directly on the skin at the level of the umbilicus in a standing position to the nearest 0.1 cm. Daily step counts were recorded using a uniaxial electrical accelerometer (Lifecorder; Kenz, Aichi). The Lifecorder was worn by participants for seven consecutive days, except during bathing.

### Hemodynamic parameters

Heart rate, brachial systolic blood pressure (SBP), diastolic blood pressure (DBP), pulse pressure (PP), and mean arterial pressure were measured using a semi-automated vascular testing device equipped with an electrocardiogram and oscillometric extremity cuffs (Form PWV/ABI: Model BP-203RPEII; Colin Medical Technology, Aichi). For protocol 2, the carotid hemodynamic parameters were evaluated as previously described.^(24)^ Briefly, the carotid arterial pressure was calibrated by equating the mean carotid and diastolic BP to the mean brachial and diastolic BP. Carotid augmentation index (AIx) was also calculated automatically as the pressure wave above its systolic shoulder, which was detected by fourth-order derivatives, divided by the carotid PP.

### Laboratory evaluations

Blood samples were obtained to determine total cholesterol, high-density lipoprotein (HDL) cholesterol, low-density lipoprotein (LDL) cholesterol, triglyceride, fasting blood glucose and cystatin C concentrations. Estimated glomerular filtration rate (eGFR) was calculated by using the novel equation proposed by the Japanese Society of Nephrology as follows: eGFR_cys_ (ml/min/1.73 m^2^) = [104 × Cystatin C^−1.019^ × 0.996^Age^ (×0.929: if female)] – 8. Urinary albumin and creatinine levels were collected in the laboratory and determined by standard methods, using spot urine samples. Concentrations of plasma uric acid were evaluated using liquid chromatography/mass spectrometry.

### Plasma XOR activity evaluation

Evaluation of plasma XOR activity was performed according to the method previously described by Murase *et al.*^(25,26)^ using liquid chromatography combined with triple quadrupole mass spectrometry. Briefly, plasma samples were purified using a Sephadex G25 column and were added to Tris buffer (pH 8.5) containing [^13^C_2_,^15^N_2_] xanthine, NAD^+^ and [^13^C_3_,^15^N_3_] uric acid. These mixtures were incubated at 37°C for 90 min. Subsequently, the mixtures were treated and production levels of [^13^C_2_,^15^N_2_] uric acid were evaluated using a liquid chromatography (NANOSPACE SI-2, Shiseido)-triple quadrupole mass spectrometer (SQ-Quantum, Thermo Fisher Scientific, Inc., MA). XOR activity was expressed as [^13^C_2_,^15^N_2_] uric acid in pmol/h/ml plasma.

### Statistical analysis

For protocol 1, the variables were expressed as the mean ± SD or frequency counts (for categorical data). Plasma XOR activity was log-transformed, obtaining a normal distribution, prior to univariate and multivariate linear regression analyses. Pearson’s correlation coefficient was used to express linear correlation between variables. Independent correlates of log-transformed plasma XOR activity were examined using a multivariate linear regression analysis with a forward stepwise procedure. To consider multicollinearity, the body mass index, visceral fat and waist circumference were entered in the three difference stepwise models, separately. For protocol 2, the variables were expressed as the mean ± SE of the mean. Mann-Whitney *U* test was used to identify differences in the baseline characteristics of the participants between the two groups. Wilcoxon signed-rank test was used to compare the difference in the baseline and post-intervention evaluations in the groups. To compare the differences in the effects on daily step counts, visceral fat, plasma XOR activity and carotid AIx throughout the intervention between the two groups, we performed the Mann-Whitney *U* test. Spearman’s rank correlation coefficients (*r*_s_) were used to express linear correlations between the changes in daily step counts and visceral fat, plasma XOR activity and carotid AIx after the 12-week intervention. For protocols 1 and 2, *p*<0.05 was considered to indicate a statistically significant difference for all comparisons. All statistical analyses were performed using SPSS software (ver. 21; IBM Corp., NY).

## Results

### Protocol 1

Characteristics of the selected subjects for cross-sectional study are presented in Table 1. A total of 94 middle-aged and older women participated in the cross-sectional study. Although the majority of subjects were post-menopausal, a few pre-menopausal women were included (*n* = 7). The mean age of participants was 60 ± 6 years, mean body mass index was 23.1 ± 2.9 kg/m^2^, mean visceral fat was 51 ± 20 cm^2^ and mean values of plasma XOR activity were 43 ± 58 pmol/h/ml plasma.

**Fig. 1:**
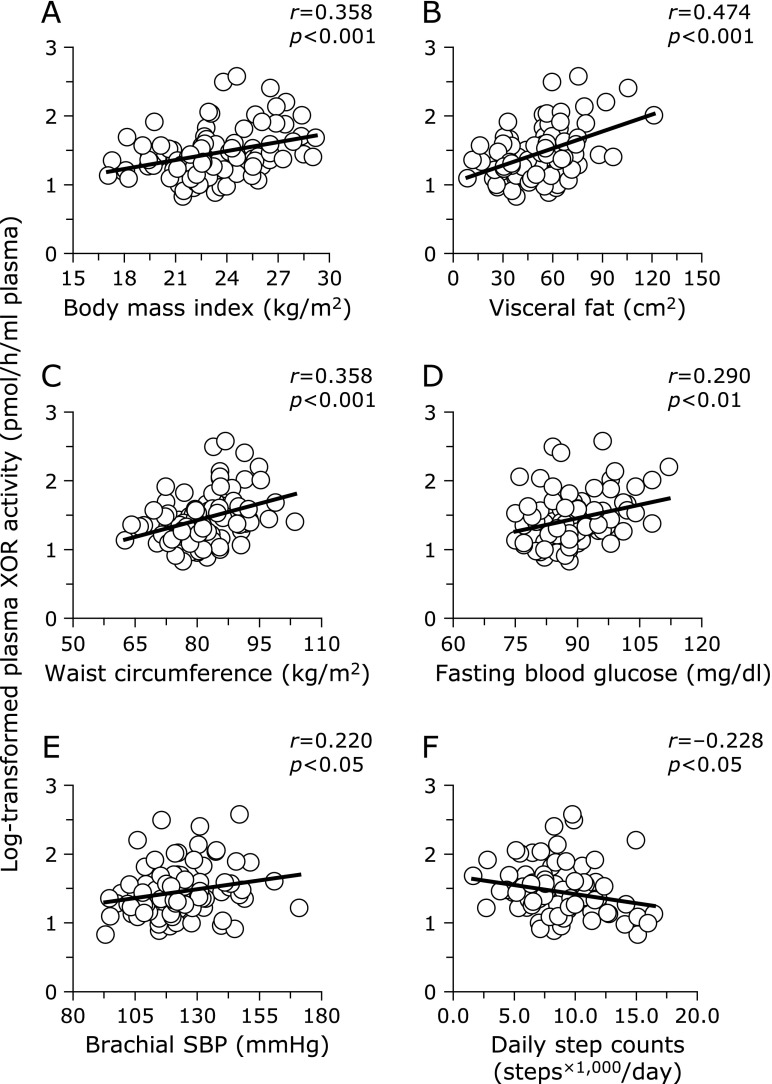
Univariate correlations between log-transformed XOR activity and various physiological parameters. XOR, xanthine oxidoreductase.

Figure 1 presents the univariate associations between log-transformed plasma XOR activity and various physiological parameters. The log-transformed plasma XOR activity was positively correlated with body mass index (*r* = 0.358; *p*<0.001; A), visceral fat (*r* = 0.474; *p*<0.001; B), waist circumference (*r* = 0.358; *p*<0.001; C), brachial SBP (*r* = 0.220; *p*<0.05; D) and fasting blood glucose (*r* = 0.290, *p*<0.01; E), and was inversely correlated with daily step counts (*r* = −0.228; *p*<0.05; F). Furthermore, the log-transformed plasma XOR activity was significantly associated with plasma uric acid levels (*r* = 0.211; *p*<0.05). Conversely, the renal function parameters, eGFR_cys_ (*r* = 0.046) and urinary albumin levels (*r* = 0.050), were not significantly associated with the log-transformed plasma XOR activity.

Table 2 presents the results of the multivariate linear regression analyses for three various stepwise models. In the initial model (Model 1), which considered potentially relevant factors and body mass index, the daily step counts were revealed to be weak but significantly independently associated with the log-transformed plasma XOR activity (β = −0.22; *p* = 0.021). In the subsequent models (Models 2 and 3), considering the visceral fat and waist circumference, the daily step counts were also capable of independently determining the log-transformed plasma XOR activity (β = −0.19; *p* = 0.040 and β = −0.20; *p* = 0.033, respectively). When the body mass index, visceral fat and waist circumference were simultaneously entered, the results were similar to that of Model 2.

### Protocol 2

Characteristics of the selected subjects before and after the intervention are presented in Table 3. Only one pre-menopausal woman was included in the exercise group. Prior to intervention, there were no significant differences between the groups for all variables. After the 12-week intervention, daily step counts were significantly increased and mean arterial pressure, brachial and carotid SBP, DBP, carotid AIx, total cholesterol and plasma XOR activity were significantly decreased in the exercise group (*p*<0.05 all). On the other hand, visceral fat and fasting blood glucose levels were significantly increased in the control group (*p*<0.05 both). Weight, body mass index, waist circumference, heart rate, triglyceride, eGFR_cys_, urinary albumin levels and plasma uric acid levels were not significantly changed after the 12-week intervention in both groups.

**Fig. 2:**
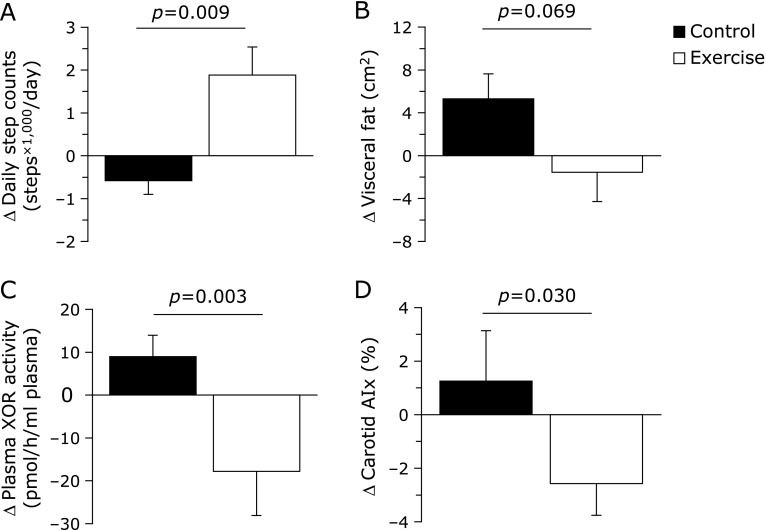
Changes in daily step counts (A), visceral fat (B), plasma XOR activity (C) and carotid AIx (D), after the 12-week aerobic exercise training. Data are presented as the mean ± SE of the mean. XOR, xanthine oxidoreductase; AIx, augmentation index.

Figure 2 presents the differences in the effects on daily step counts, visceral fat, plasma XOR activity and carotid AIx throughout the intervention between the two groups. Significant differences were demonstrated in the changes in daily step counts (*p* = 0.009), plasma XOR activity (*p* = 0.003) and carotid AIx (*p* = 0.030) were observed between the two groups. The alterations in visceral fat varied between the two groups; however, this difference was not significant (*p* = 0.069).

**Fig. 3:**
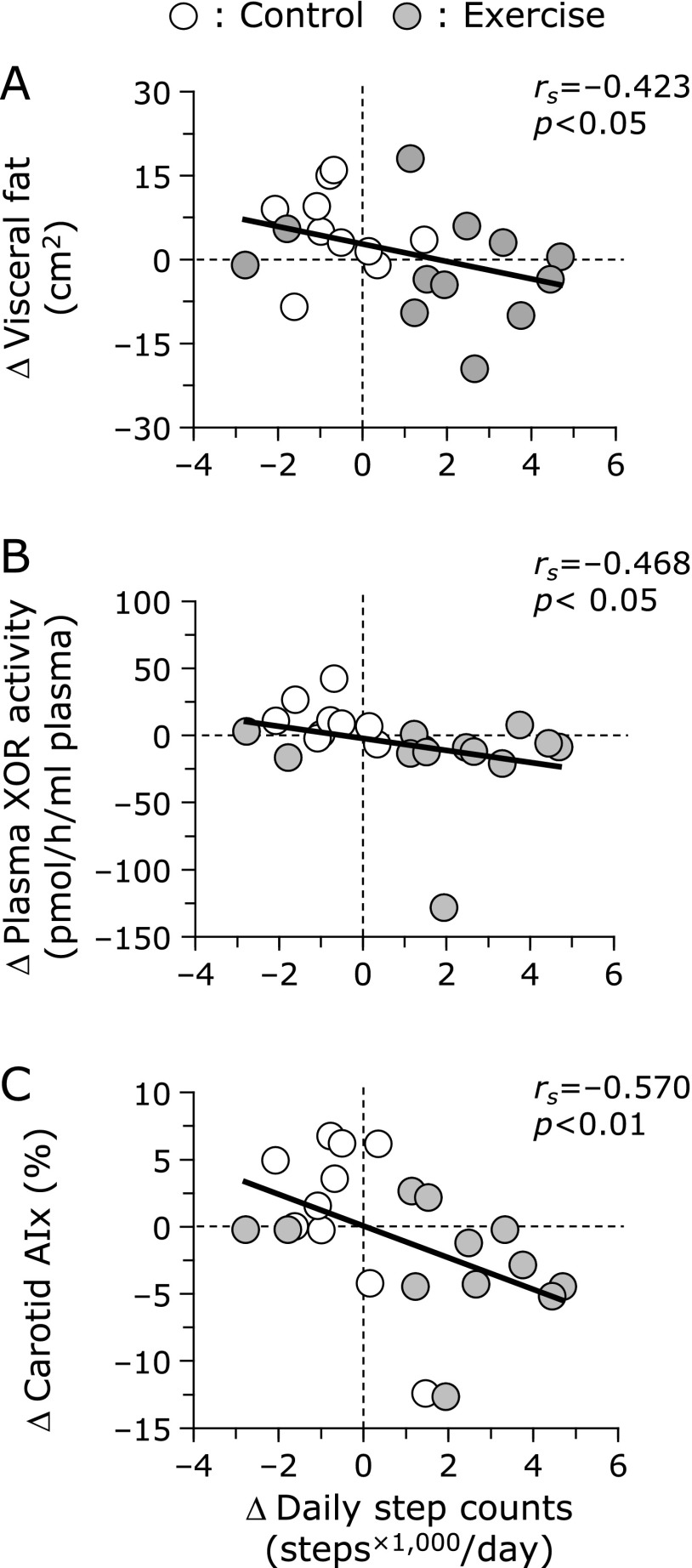
Associations between the changes in daily step counts and visceral fat (A), plasma XOR activity (B) and carotid AIx (C), after the 12-week aerobic exercise training. XOR, xanthine oxidoreductase; AIx, augmentation index.

Figure 3 presents the association between the changes in daily step counts and visceral fat, plasma XOR activity and carotid AIx after the 12-week intervention. The changes in daily step counts were inversely correlated with the changes in visceral fat (*r*_s_ = −0.423; *p*<0.05), plasma XOR activity (*r*_s_ = −0.468; *p*<0.05) and carotid AIx (*r*_s_ = −0.570; *p*<0.01).

## Discussion

The salient findings of the present study were as follows: Firstly, in the cross-sectional study, the plasma XOR activity was associated with various physiological parameters, including visceral fat and daily step counts, in middle-aged and older women. Furthermore, the association between plasma XOR activity and daily step counts remained significant after consideration of confounders, including the other physiological parameters. Secondly, in the interventional study, the plasma XOR activity was significantly decreased after the 12-week aerobic exercise training; its alterations were inversely associated with the changes in daily step counts in middle-aged and older women. These findings indicated that, even in healthy middle-aged and older women, the plasma XOR activity was associated with visceral fat accumulation and lack of exercise, and it was likely to be decreased by aerobic exercise training. To the best our knowledge, this is the first study to investigate the effect of habitual exercise on XOR activity, and suggests that habitual exercise is an effective intervention to decrease the plasma XOR activity in middle-aged and older women.

In addition to several disease-associated studies,^(9–11)^ the most recent cross-sectional study has demonstrated that the plasma XOR activity was associated with insulin resistance and serum uric acid levels in healthy young individuals.^(27)^ This finding suggests that the plasma XOR activity appears to be related to various physiological changes even if not in the state of chronic disease. We, therefore, investigated the association between plasma XOR activity and some physiological parameters in middle-aged and older women who are healthy but has age- and menopause-associated CVD risks. Even when various physiological parameters were within the normal range, the plasma XOR activity was significantly associated with body mass index, visceral fat, waist circumference, brachial SBP and fasting blood glucose levels (i.e., the parameters associated with the onset of CVD) in middle-aged and older women. These results indicate that the increase in plasma XOR activity is associated with various physiological changes, including visceral fat accumulation and increased blood pressure and blood glucose in middle-aged and older women. Keeping the low levels of plasma XOR activity, accordingly, may be important for prevention of CVD development in middle-aged and older women.

Furthermore, the cross-sectional investigation of the present study revealed another important finding; the plasma XOR activity was independently associated with daily step counts in middle-aged and older women. We initially hypothesized that the reduction of visceral fat by habitual exercise led to decrease plasma XOR activity; however, the result obtained from the cross-sectional investigation indicated that habitual exercise might decrease plasma XOR activity without alteration to visceral fat. Therefore, in protocol 2, we investigated the effect of 12-week aerobic exercise training on plasma XOR activity in middle-aged and older women. In the interventional study, the 12-week aerobic exercise training significantly increased daily step counts; however, visceral fat was not significantly altered. This may be because the effect of exercise on visceral fat is not sufficient as the subject’s baseline visceral fat is comparatively small (54 ± 8 cm^2^). However, the plasma XOR activity was significantly decreased after the 12-week aerobic exercise training. Partially contrary to our hypothesis, the results of the cross-sectional and interventional study suggested that the plasma XOR activity might be decreased by habitual exercise without significant changes in visceral fat. XOR expression has previously been observed in various organs, including the liver, lung, heart and kidney, in addition to vascular (i.e., endothelial cells) and adipose tissues.^(5)^ Therefore, the decreased XOR activity in other organs by aerobic exercise training may have induced the decrease in plasma XOR activity in the present study.

It remains to be elucidated whether the decrease in plasma XOR activity by habitual exercise induces the prevention of CVD onset, as a prospective observational study has not yet been performed. Although all we can do is just to speculate, we propose the clinical significance of the decrease in plasma XOR activity by habitual exercise from the present some results. In the interventional study, the 12-week aerobic exercise training decreased the brachial and carotid blood pressure similar to the plasma XOR activity. Furthermore, the carotid AIx, which is more strongly associated with CVD risks,^(28,29)^ also decreased after the 12-week aerobic exercise training and its changes were inversely associated with the changes in daily step counts. In addition to these results, the changes in carotid AIx positively correlated with the changes in plasma XOR activity (*r*_s_ = –0.434; *p*<0.05). Taken together, after the 12-week aerobic exercise training, the carotid AIx appeared to have decreased with the degree of decrease in plasma XOR activity. These results indicate that the decrease in plasma XOR activity by habitual exercise may be associated with decreasing the CVD risks in middle-aged and older women.

There are a number of noteworthy limitations to the present study: i) this was a single-center study that included a relatively small sample size; ii) the potential mechanisms underlying the effects of habitual exercise on plasma XOR activity were not investigated; iii) we could not evaluate a number of oxidative stress and inflammation markers, which were likely to reduce with the decrease in plasma XOR activity; iv) the effects of habitual exercise on plasma XOR activity in men with CVD risks higher than that of women remains unidentified; v) the association between the decreased plasma XOR activity and the prevention of CVD event was not demonstrated in the present study because of the observational nature of this investigation. Accordingly, further multi-center and prospective cohort studies with larger sample sizes including men, and experimental basic studies are required to identify the clinical significance and potential mechanisms underlying the decrease in plasma XOR activity by habitual exercise.

In conclusion, the plasma XOR activity was associated with various physiological parameters, including visceral fat and daily step counts, and it was decreased by the 12-week aerobic exercise training in middle-aged and older women. These findings indicate that habitual exercise is an effective intervention to decrease the plasma XOR activity and may induce prevention of the onset of CVD in middle-aged and older women. We believe that the present study provides one of the physiological mechanisms for preventing the onset of CVD by habitual exercise in middle-aged and older women.

## Funding

This research did not receive any specific grant from funding agencies in the public, commercial, or not-for-profit sectors.

## Figures and Tables

**Table 1:** Characteristics of selected subjects for cross-sectional study

Variable	Total
*n*	94
Postmenopausal women, *n* (%)	87 (93)
Age, years	60 ± 6
Height, cm	156 ± 6
Weight, kg	56.5 ± 7.8
Body mass index, kg/m^2^	23.1 ± 2.9
Visceral fat, cm^2^	51 ± 20
Waist circumference, cm	82 ± 8
Daily step counts, steps^×1^^,^^000^/day	8.7 ± 3
Heart rate, bpm	61 ± 7
Mean arterial pressure, mmHg	90 ± 11
Brachial systolic blood pressure, mmHg	123 ± 15
Brachial diastolic blood pressure, mmHg	74 ± 9
Total cholesterol, mg/dl	226 ± 32
High-density lipoprotein cholesterol, mg/dl	65 ± 15
Low-density lipoprotein cholesterol, mg/dl	140 ± 29
Triglyceride, mg/dl	90 ± 48
Fasting blood glucose, mg/dl^§^	89 ± 8
eGFR_cys_ ml/min/1.73 m^2^	88 ± 13
Urinary albumin levels mg/g creatinine^#^	7.9 ± 5.3
Plasma uric acid levels, µM	201 ± 44
Plasma XOR activity, pmol/h/ml plasma	43 ± 58

Data are shown as the mean ± SD or frequency counts (%), as appropriate. ^§^Data were available in 93 subjects. ^#^Data were available in 86 subjects. eGFR_cys_, estimated glomerular filtration rate by calculated serum cystatin C levels. XOR, xanthine oxidoreductase.

**Table 2:** Multivariate linear regression models for log-transformed plasma XOR activity

Variable	B ± SEM (×10)	β	*p*
**Model 1: body mass index and covariates**^§^** (*R***^**2**^** = 0.218; *****p*****<0.001)**			
Body mass index, kg/m^2^	0.036 ± 0.012	0.3	0.003
Daily step counts, steps^×1^^,^^000^/day	−0.025 ± 0.011	−0.22	0.021
Fasting blood glucose, mg/dl	0.009 ± 0.004	0.2	0.04
**Model 2: visceral fat and covariates**^§^** (*R***^**2**^** = 0.302; *****p*****<0.001)**			
Visceral fat, cm^2^	0.007 ± 0.002	0.42	<0.001
Fasting blood glucose, mg/dl	0.009 ± 0.004	0.21	0.023
Daily step counts, steps^×1^^,^^000^/day	−0.021 ± 0.01	−0.19	0.04
**Model 3: waist circumference and covariates**^§^** (*R***^**2**^** = 0.223; *****p*****<0.001)**			
Waist circumference, cm	0.014 ± 0.004	0.3	0.002
Fasting blood glucose, mg/dl	0.01 ± 0.004	0.22	0.025
Daily step counts, steps^×1^^,^^000^/day	−0.023 ± 0.011	−0.2	0.033

^§^Covariates included in the multiple linear regression models were brachial systolic blood pressure, high-density lipoprotein cholesterol, low-density lipoprotein cholesterol, triglyceride, fasting blood glucose, estimated glomerular filtration rate, plasma uric acid levels and daily step counts. XOR, xanthine oxidoreductase.

**Table 3:** Characteristics of selected subjects for interventional study

Variable	Control (*n* = 10)		Exercise (*n* = 12)	
	Before	After	Before	After
Age, years	61 ± 1	—	60 ± 1	—
Height, cm	160 ± 4	—	157 ± 2	—
Weight, kg	55.3 ± 3.2	55.6 ± 3.2	58.2 ± 1.7	58 ± 1.7
Body mass index, kg/m^2^	21.6 ± 0.9	21.7 ± 0.9	23.7 ± 0.7	23.6 ± 0.7
Visceral fat, cm^2^	48 ± 5	54 ± 6*	54 ± 8	53 ± 7
Waist circumference, cm	80 ± 3	82 ± 3	84 ± 2	85 ± 2
Daily step counts, steps^×1^^,^^000^/day	9.1 ± 1	8.5 ± 0.8	8.4 ± 0.8	10.3 ± 0.6*
Heart rate, bpm	59 ± 2	58 ± 8	61 ± 2	60 ± 2
Mean arterial pressure, mmHg	84 ± 3	83 ± 2	87 ± 2	83 ± 2*
Brachial systolic blood pressure, mmHg	113 ± 3	112 ± 3	117 ± 3	112 ± 2*
Brachial diastolic blood pressure, mmHg	69 ± 3	69 ± 2	71 ± 2	68 ± 2*
Carotid systolic blood pressure, mmHg	104 ± 3	104 ± 3	108 ± 3	103 ± 2*
Carotid diastolic blood pressure, mmHg	69 ± 3	69 ± 3	72 ± 2	68 ± 2*
Carotid augmentation index, %	28 ± 2	30 ± 2	24 ± 2	21 ± 2*
Total cholesterol, mg/dl	219 ± 6	215 ± 11	231 ± 10	216 ± 10*
Triglyceride, mg/dl	85 ± 12	82 ± 12	109 ± 18	114 ± 22
Fasting blood glucose, mg/dl	88 ± 3	92 ± 2*	91 ± 3	92 ± 2
eGFR_cys_, ml/min/1.73 m^2^	103 ± 5	102 ± 4	107 ± 6	104 ± 4
Urinary albumin levels, mg/g creatinine	11.2 ± 3.1	12.1 ± 5.2	6.9 ± 1.2	6.9 ± 1.1
Plasma uric acid levels, µM	197 ± 10	204 ± 8	186 ± 17	184 ± 15
Plasma XOR activity, pmol/h/ml plasma	24 ± 5	33 ± 8	58 ± 21	41 ± 14*

Data are shown as the mean ± SE of the mean. *p<0.05 vs Before. eGFR_cys_, estimated glomerular filtration rate by calculated serum cystatin C levels. XOR, xanthine oxidoreductase.

## Abbreviations

AIx: augmentation index
CVD: cardiovascular disease
XOR: xanthine oxidoreductase
